# Case report of extensive embolic complications in culture-negative mitral valve endocarditis due to *Rothia dentocariosa*

**DOI:** 10.1093/ehjcr/ytag097

**Published:** 2026-02-06

**Authors:** Hing Yin Wilson Lam

**Affiliations:** Department of Medicine, Yan Chai Hospital, 7–11 Yan Chai Street, Tsuen Wan, New Territories, Hong Kong

**Keywords:** Culture-negative infective endocarditis, *Rothia dentocariosa*, Next-generation sequencing

## Abstract

**Background:**

Culture-negative infective endocarditis presents significant diagnostic and therapeutic challenges, especially in patients with systemic embolic complications.

**Case summary:**

A middle-aged man with a history of intravenous drug use presented with fever, new apical systolic murmur, and widespread embolic phenomena affecting the brain, spleen, kidneys, and lower limb. Transthoracic echocardiography revealed a large posterior mitral leaflet vegetation with moderate–severe mitral regurgitation. Blood and tissue cultures were negative. Next-generation sequencing (NGS) of an aspirated calf muscle abscess identified *Rothia dentocariosa*. Despite prolonged antimicrobial therapy and multidisciplinary care, persistent vegetations and high embolic risk prompted mitral valve replacement after interval stabilization of intracranial haemorrhages. He recovered without recurrent emboli and with stable prosthetic valve function.

**Discussion:**

Advanced molecular diagnostics such as NGS can be crucial for identifying pathogens in culture-negative endocarditis.

Multidisciplinary collaboration and individualized surgical timing are important for achieving optimal outcomes in complex cases with neurological complications.

Learning points

- Blood culture-negative infective endocarditis requires a structured diagnostic work-up with repeat blood cultures, targeted serology, and molecular testing (e.g. 16S rRNA PCR/NGS) in line with the European Society of Cardiology guidelines.
- In infective endocarditis complicated by intracerebral haemorrhage, the timing of valve surgery should be individualized to balance embolic risk against neurological deterioration.

## Introduction

Blood culture-negative infective endocarditis (BCNIE) accounts for up to 20% of infective endocarditis (IE) cases and is associated with delayed diagnosis and high mortality.^1^  *Rothia dentocariosa*, a Gram-positive coccobacillus, is a rare cause of IE, most often linked to dental disease or intravenous drug use (IVDU). Next-generation sequencing (NGS) can assist diagnosis when conventional microbiology is unrevealing.

We report a case of BCNIE with extensive embolic complications in which *R. dentocariosa* was identified by NGS from a peripheral abscess. This case illustrates the diagnostic value of molecular techniques in BCNIE and the complexity of surgical timing in a patient with large vegetations, multisystem embolization and intracerebral haemorrhage.

## Summary figure

**Figure:**
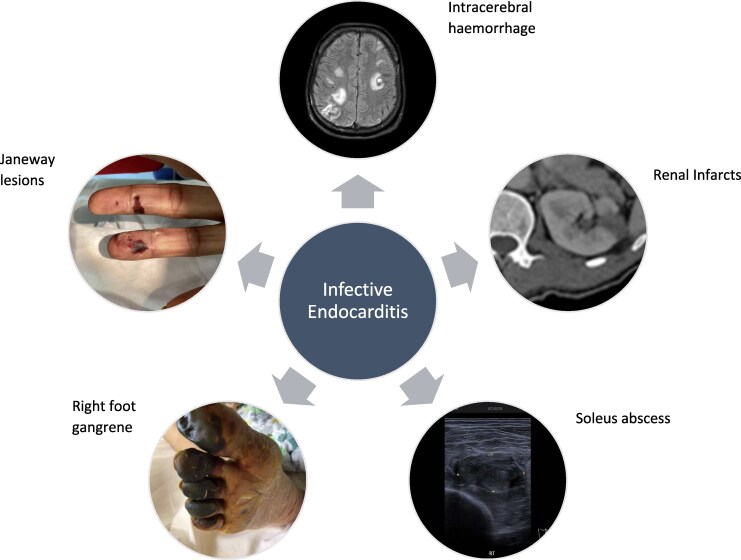
Blood culture-negative infective endocarditis (mitral valve) with extensive embolic complications

## Case presentation

A 58-year-old male smoker with previous IVDU was found on the floor at home and brought to the hospital with fever and altered mental state. He had no known chronic illness or cardiac disease. His history was limited, and he denied chest pain or dyspnoea.

On admission (Day 0), his temperature was 38°C; blood pressure 116/66 mmHg, pulse 97/min, and oxygen saturation 97% on room air. He required no vasopressors or respiratory support. Examination revealed poor oral hygiene, a new apical systolic murmur, and peripheral stigmata of IE (Osler’s nodes and Janeway lesions).

Initial investigations (*Table 1*) showed leucocytosis and elevated high-sensitivity troponin I (peak 7750 ng/L). A 12-lead electrocardiogram showed sinus tachycardia without ischaemic changes. Chest radiography was unremarkable. Urine toxicology was positive for methamphetamine.

**Table 1:** Serial laboratory parameters and antimicrobial therapy during the clinical course

Hospital day/event	WBC (×10⁹/L) (3.7–9.2)	Hb (g/dL) (13.4–17.0)	Plt (×10⁹/L) (145–370)	Creatinine (µmol/L) (65–109)	Urea (mmol/L) (3.1–7.8)	ALT (U/L) (<53)	ALP (U/L) (43–105)	Total bilirubin (µmol/L) (<22)	C-reactive protein (mg/L) (<5.0)	ESR (mm/h) (<32)	Antibiotic regimen*^a^*
Day 0 (admission)	25.4	12.5	235	96	7.5	114	200	32			Day 0–1: piperacillin–tazobactam Day 1–2: amoxicillin–clavulanate Day 2–4: ceftriaxone
Day 1									208	–	
Day 2									–		
Day 4									253	57	Day 4–6: ceftriaxone + ampicillin Day 6–13: ceftriaxone + ampicillin + gentamicin Day 13–18: ceftriaxone + ampicillin
Day 6 (first TTE)									153	–	
Day 13									120	–	
Day 17									109	–	Day 18–82: ceftriaxone
Day 18									–		
Day 35									38	–	
Day 38									28	–	
Day 54									26	76	
Day 60									123	89	
Day 62									67	87	
Day 63									51	79	
Day 67									41	63	
Day 74									153	101	
Day 76									86	64	
Day 81									34	102	
Day 82											From Day 82: antibiotics stopped

*Reference intervals are quoted in parentheses.*

ALP, alkaline phosphatase; ALT, alanine aminotransferase; ESR, erythrocyte sedimentation rate; Hb, haemoglobin; Plt, platelet count; TTE, transthoracic echocardiogram; WBC, white blood cell count.

^*a*^ *Doses of antibiotic regimen*: piperacillin–tazobactam 4.5 g Q8H i.v., amoxicillin–clavulanate 1.2 g Q8H i.v., ceftriaxone 1 g Q12H i.v., ampicillin 2 g Q4H i.v., and gentamicin 80 mg Q8H i.v.

**Figure 1:**
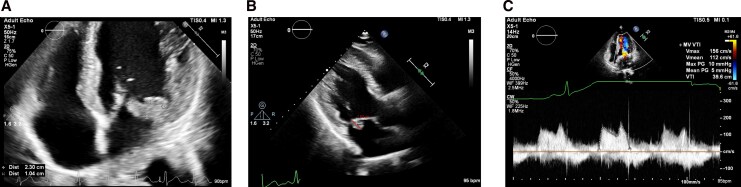
(*A*) Baseline transthoracic echocardiography showed posterior mitral valve leaflet vegetation 2.3 × 1.0 cm. (*B*) Transthoracic echocardiography after complete 11-week ceftriaxone showed persistent infective endocarditis over mitral valve > 1 cm. (*C*) Transthoracic echocardiography of post-mitral valve (MV) replacement showed MV prosthesis *in situ*, and there is no obvious leakage seen. The mean pressure gradient across MV prosthesis is 5 mmHg.

Transthoracic echocardiography (TTE) on Day 6 (*Figure 1A*; Supplementary material online, *Video S1A*/*B*) showed a mildly enlarged left atrium, preserved biventricular systolic function, and a 2.3 × 1.0 cm mobile vegetation on the posterior mitral leaflet with moderate–severe mitral regurgitation (MR). Right ventricular systolic pressure was 26 mmHg. No pericardial effusion was present.

Multiple blood and fungal cultures were negative. Serology for atypical pathogens (including *Bartonella* spp., *Brucella* spp., *Coxiella burnetii*, *Chlamydia* spp., and *Mycoplasma pneumoniae*) was negative. Autoimmune screening, complement levels, and serum protein electrophoresis were unremarkable. Dental assessment found no active source, and there had been no recent dental procedure.

Empirical antibiotics were commenced on Day 0 and adjusted following infectious diseases review (*Table 1*). Therapy was consolidated to ceftriaxone monotherapy for a total of 11 weeks. He remained afebrile and the leucocytosis resolved, but C-reactive protein fluctuated between 26 and 153 mg/L (normal < 5 mg/L).

The hospital course was complicated by multifocal embolic events.

**Figure 2:**
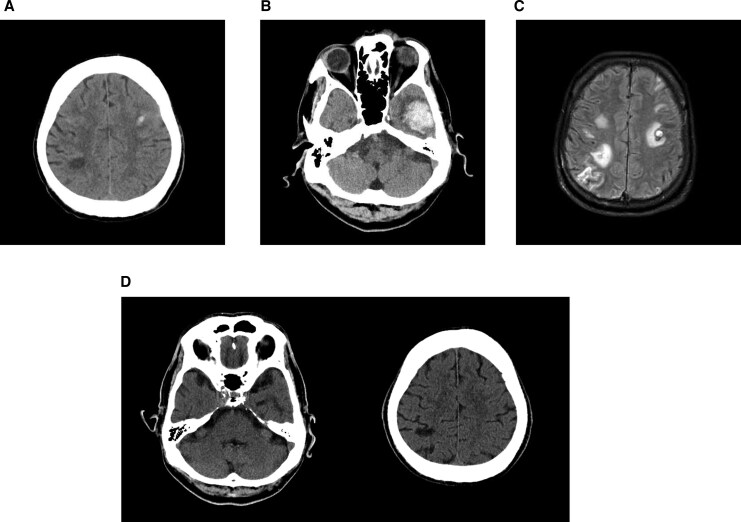
(*A*) Brain CT showed right parietal hypodensity and a small left frontoparietal intracerebral haemorrhage. (*B*) Repeat CT on follow-up revealed a new large left temporal lobe haemorrhage. (*C*) MRI (T2 fluid-attenuated inversion recovery (FLAIR)) demonstrated multiple rim-enhancing lesions. (*D*) Subsequent brain CT showed resolution of intracerebral haemorrhage.

*Neurological:* Within 24 h, he developed dysarthria and facial asymmetry. Computer tomography (CT) of brain on Day 1 (*Figure 2A*) showed right parietal hypodensities and a small left frontoparietal intracerebral haemorrhage (ICH). Repeat CT on Day 3 (*Figure 2B*) showed a new large left temporal ICH with additional small bilateral frontal haemorrhages. Magnetic resonance imaging (MRI) on Day 14 (*Figure 2C*) demonstrated multiple rim-enhancing lesions, including one with acute haemorrhage, consistent with septic emboli and haemorrhagic transformation. Follow-up CT on Day 84 (*Figure 2D*) confirmed resolution of haemorrhage and maturation of infarcts. His neurological deficits resolved to baseline. CT angiography showed no mycotic aneurysm, and neurosurgical intervention was not required.

**Figure 3:**
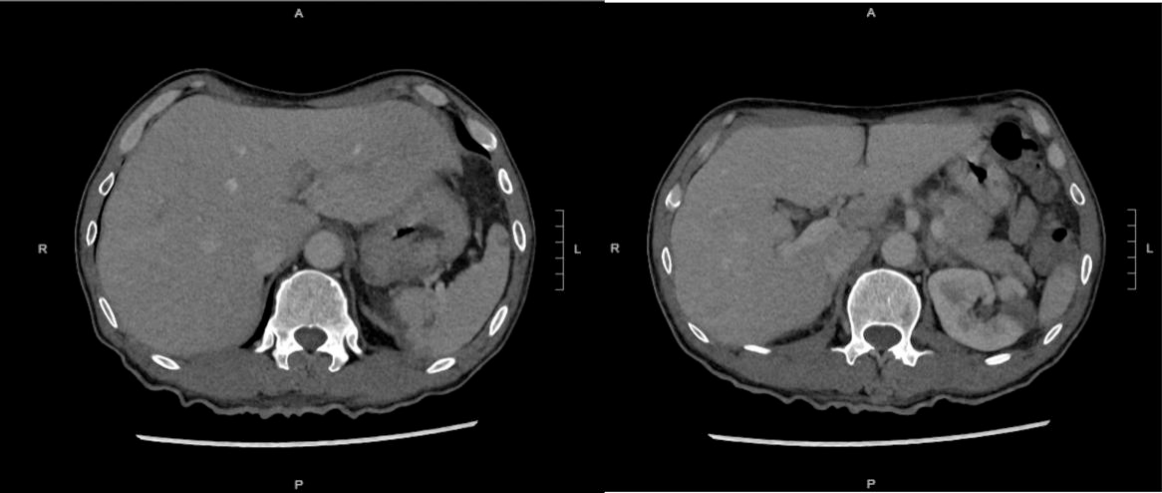
CT of the abdomen and pelvis showed splenic and renal infarcts.

*Systemic:* Contrast-enhanced CT of the abdomen and pelvis demonstrated multiple splenic and renal infarcts without abscess formation (*Figure 3*), consistent with systemic embolization.

**Figure 4:**
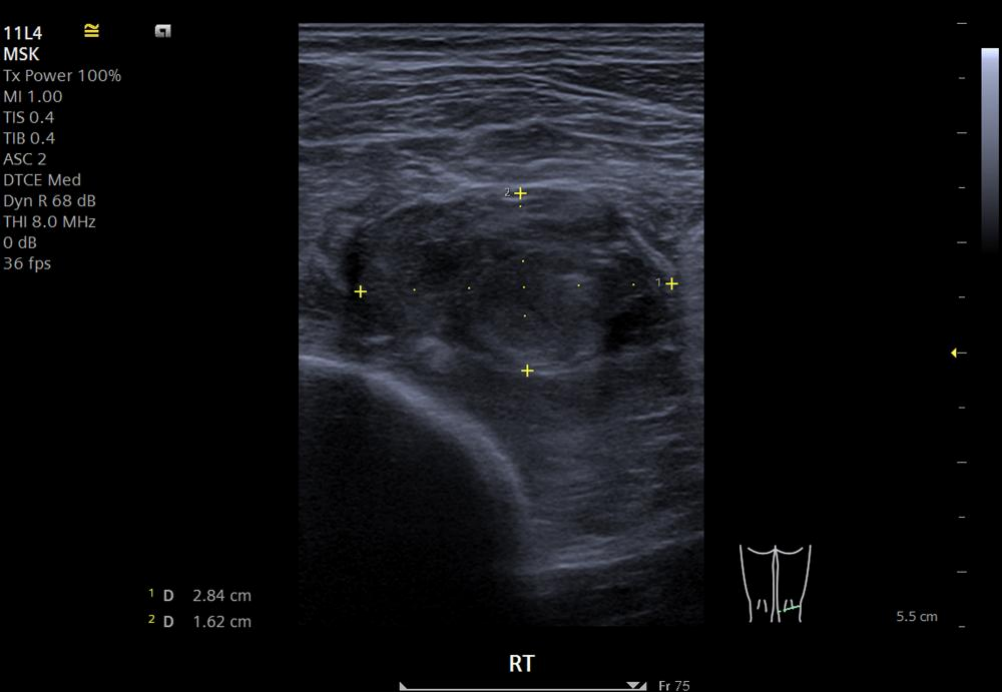
Ultrasound-guided drainage of right soleus muscle abscess.

*Musculoskeletal and vascular:* By Week 3, he developed right foot gangrene. Lower limb CT angiography showed rim-enhancing abscesses in the right flexor hallucis longus and soleus muscles (largest 2.2 cm) and a tibioperoneal trunk pseudoaneurysm (3.4 × 2.9 × 5.5 cm). The posterior tibial artery reconstituted distally via collateral vessels. Ultrasound-guided aspiration and drainage of the calf abscess were performed on Day 19 (*Figure 4*). Conventional cultures were negative, but NGS of the aspirate identified *R. dentocariosa* 16S rRNA gene. As limb perfusion was stable, vascular and orthopaedic teams advised conservative management of the pseudoaneurysm and dry gangrene with close follow-up.

Despite prolonged antibiotics, repeat TTE on Day 79 showed a persistent mobile vegetation > 1 cm and ongoing moderate–severe MR (*Figure 1B*; Supplementary material online, *Video S2*). After multidisciplinary review, and once interval neuroimaging confirmed ICH resolution, surgical mitral valve replacement with a 29 mm bioprosthesis was performed ∼3 months after admission. Preoperative transoesophageal echocardiography (TEE) confirmed a 1.4 cm mobile vegetation on P2 with a flail segment and severe eccentric MR. Intraoperatively, two ∼1 cm vegetations were found on P2 with ruptured chordae at A1, precluding valve repair. The excised valve was culture negative. Postoperative TTE (*Figure 1C*; Supplementary material online, *Video S3*) showed a well-seated prosthesis with no paravalvular leak.

Recovery was uneventful, and at 1-month follow-up, he was asymptomatic with normalized inflammatory markers.

## Discussion

This case highlights challenges in BCNIE, including diagnostic uncertainty despite extensive investigations, the value of molecular diagnostics, the high embolic risk of large mobile mitral vegetations, and the need to individualize surgical timing after ICH.

The 2023 European Society of Cardiology (ESC) guidelines recommend a structured BCNIE pathway incorporating repeat blood cultures, targeted serology, pathogen-specific polymerase chain reaction (PCR), and consideration of non-bacterial thrombotic endocarditis.^2^ Despite broad testing, conventional microbiology and serology were unrevealing in our patient.

*Rothia dentocariosa* is a rare cause of IE with few cases reported.^3^ Risk factors include periodontal disease, recent dental manipulation, and IVDU.^4^ In this case, NGS of a drained calf abscess identified *R. dentocariosa*, providing a plausible aetiology when standard cultures were negative. A limitation is the absence of molecular testing on the excised valve, which would have strengthened causal attribution.

There are no guideline-specific antibiotic recommendations for *R. dentocariosa* endocarditis, and treatment is informed by case reports and susceptibility patterns. Reported regimens include penicillin for 4–6 weeks with or without gentamicin, and ceftriaxone monotherapy has also been described.^5^ In this patient, the NGS result supported prolonged ceftriaxone in the context of persistent vegetation, extensive embolization, and fluctuating inflammatory markers.

The extent of embolization to the brain, abdominal organs, and peripheral vasculature is consistent with the recognized embolic risk associated with large, mobile left-sided vegetations. This reinforces the importance of early multimodality imaging and coordinated multidisciplinary care.

The major management dilemma was surgical timing. The ESC guidelines recommend urgent surgery (within 3–5 days) for patients with vegetations ≥ 1 cm following an embolic event to reduce the risk of further embolization.^2^ In the presence of ICH, however, they give a Class IIa recommendation to defer surgery for >1 month where possible, with frequent clinical and imaging reassessment to minimize neurological risk. In our patient, persistent large vegetation and ongoing inflammatory activity suggested medical therapy alone was insufficient, yet early cardiopulmonary bypass carried substantial neurological risk. Delayed surgery with prolonged antimicrobial therapy and serial neuroimaging enabled mitral valve replacement once ICH had resolved.

Percutaneous vegetation aspiration has emerged as a potential bridge to surgery in selected high-risk patients,^6^ but experience is predominantly in right-sided or device-related infection. Evidence for left-sided disease remains limited, and it is not standard care. Potential risks include systemic embolization, valve or chordal injury, and intraprocedural anticoagulation-related bleeding, with uncertain long-term outcomes. Given severe MR, persistent large vegetation, and intraoperative evidence of extensive leaflet and chordal destruction, definitive surgical valve replacement rather than repair was the most appropriate strategy in this patient.

## Conclusion

Blood culture-negative infective endocarditis due to rare organisms may require molecular diagnostics such as NGS when conventional testing is unrevealing. In patients with large mitral vegetations complicated by intracranial haemorrhage, surgical timing should be individualized through multidisciplinary assessment balancing embolic and neurological risks.

## Lead author biography


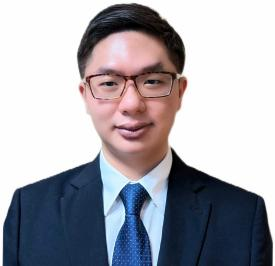


Hing Yin Wilson Lam graduated from the University of Hong Kong. He is currently working in the Department of Medicine, Yan Chai Hospital. He is interested in interventional cardiology and percutaneous coronary intervention.

## Supplementary Material

ytag097_Supplementary_Data

## Data Availability

The data underlying this article are not publicly available in order to protect patient privacy but may be shared in de-identified form on reasonable request from the corresponding author.

## Notes

**Consent:** Written informed consent for publication of this case report and any accompanying images was obtained from the patient in accordance with the COPE guidelines.
